# Supplementary material for: Modeling glioblastoma heterogeneity as a dynamic network of cell states
Source: Mol Syst Biol. 2021 Sep 16;17(9):e10105. doi: 10.15252/msb.202010105 (PMC8444284; doi:10.15252/msb.202010105)
Supplement: Supplementary file 6 — Source Data for Figure 5 [file MSB-17-e10105-s004.zip › Figure5A_sourcedata/GSEA_3071/hallmarks_stateI.GseaPreranked.1623742531165/HALLMARK_COAGULATION.html]

Details for gene set HALLMARK\_COAGULATION[GSEA]

|  || Dataset | state13071 |
| Phenotype | NoPhenotypeAvailable |
| Upregulated in class | na\_pos |
| GeneSet | HALLMARK\_COAGULATION |
| Enrichment Score (ES) | 0.5039766 |
| Normalized Enrichment Score (NES) | 2.3956976 |
| Nominal p-value | 0.0 |
| FDR q-value | 3.4444444E-4 |
| FWER p-Value | 0.001 |
Table: GSEA Results Summary

  

Fig 1: Enrichment plot: HALLMARK\_COAGULATION      
 Profile of the Running ES Score & Positions of GeneSet Members on the Rank Ordered List

  

| PROBE | GENE SYMBOL | GENE\_TITLE | RANK IN GENE LIST | RANK METRIC SCORE | RUNNING ES | CORE ENRICHMENT || 1 | FN1 |  |  | 11 | 1.769 | 0.0583 | Yes |
| 2 | MMP14 |  |  | 27 | 1.526 | 0.1070 | Yes |
| 3 | CFH |  |  | 29 | 1.523 | 0.1596 | Yes |
| 4 | TIMP1 |  |  | 47 | 1.381 | 0.2027 | Yes |
| 5 | LRP1 |  |  | 84 | 1.160 | 0.2326 | Yes |
| 6 | F2RL2 |  |  | 87 | 1.137 | 0.2716 | Yes |
| 7 | SPARC |  |  | 106 | 1.067 | 0.3034 | Yes |
| 8 | CTSB |  |  | 129 | 0.964 | 0.3306 | Yes |
| 9 | FBN1 |  |  | 138 | 0.944 | 0.3611 | Yes |
| 10 | SH2B2 |  |  | 145 | 0.918 | 0.3913 | Yes |
| 11 | ITGA2 |  |  | 194 | 0.839 | 0.4066 | Yes |
| 12 | PRSS23 |  |  | 264 | 0.721 | 0.4118 | Yes |
| 13 | GSN |  |  | 265 | 0.720 | 0.4368 | Yes |
| 14 | MMP2 |  |  | 284 | 0.697 | 0.4558 | Yes |
| 15 | S100A13 |  |  | 306 | 0.665 | 0.4729 | Yes |
| 16 | BMP1 |  |  | 346 | 0.631 | 0.4836 | Yes |
| 17 | LAMP2 |  |  | 352 | 0.629 | 0.5040 | Yes |
| 18 | CTSO |  |  | 472 | 0.532 | 0.4882 | No |
| 19 | CPQ |  |  | 488 | 0.523 | 0.5020 | No |
| 20 | HTRA1 |  |  | 555 | 0.477 | 0.4996 | No |
| 21 | THBS1 |  |  | 613 | 0.446 | 0.4987 | No |
| 22 | FURIN |  |  | 663 | 0.426 | 0.4994 | No |
| 23 | TIMP3 |  |  | 773 | 0.391 | 0.4816 | No |
| 24 | CSRP1 |  |  | 921 | 0.345 | 0.4512 | No |
| 25 | RAPGEF3 |  |  | 1028 | 0.322 | 0.4319 | No |
| 26 | LGMN |  |  | 1031 | 0.321 | 0.4425 | No |
| 27 | ITGB3 |  |  | 1102 | 0.306 | 0.4330 | No |
| 28 | ISCU |  |  | 1266 | 0.274 | 0.3956 | No |
| 29 | USP11 |  |  | 1522 | -0.260 | 0.3311 | No |
| 30 | RABIF |  |  | 1772 | -0.290 | 0.2695 | No |
| 31 | A2M |  |  | 1808 | -0.295 | 0.2696 | No |
| 32 | ADAM9 |  |  | 2063 | -0.328 | 0.2079 | No |
| 33 | CD9 |  |  | 2444 | -0.400 | 0.1123 | No |
| 34 | PLAT |  |  | 2501 | -0.415 | 0.1106 | No |
| 35 | P2RY1 |  |  | 2751 | -0.486 | 0.0558 | No |
| 36 | CAPN5 |  |  | 2880 | -0.531 | 0.0374 | No |
| 37 | SERPINE1 |  |  | 3050 | -0.634 | 0.0108 | No |
| 38 | DUSP6 |  |  | 3227 | -0.816 | -0.0116 | No |
| 39 | CLU |  |  | 3424 | -1.329 | -0.0219 | No |
| 40 | PDGFB |  |  | 3428 | -1.343 | 0.0239 | No |
Table: GSEA details [plain text format]

  

Fig 2: HALLMARK\_COAGULATION: Random ES distribution      
 Gene set null distribution of ES for **HALLMARK\_COAGULATION**
